# Cow Milk Risk Factors Associated With Bacterial Contaminations Along Dairy Value Chain in Lushoto and Handeni Districts, Tanzania

**DOI:** 10.1002/vms3.71034

**Published:** 2026-06-16

**Authors:** Fortunate Shija, Hezron Emmanuel Nonga

**Affiliations:** ^1^ Kilimanjaro Centre for Community Ophthalmology Good Samaritan Foundation Moshi Tanzania; ^2^ Department of Veterinary Medicine and Public Health Sokoine University of Agriculture, College of Veterinary Medicine and Biomedical Sciences Chuo Kikuu Morogoro Tanzania

**Keywords:** microbial contaminations, microbial safety, milk‐borne bacteria, milk quality, unhygienic conditions

## Abstract

**Background:**

Milk and milk products are of high nutritional value but mishandling of any kind along the value chain leads to contaminations that shorten shelf life and pose public health threats.

**Objectives:**

To assess the milk handling practices, bacterial contaminations and determine selected milk‐borne zoonotic pathogens along the dairy value chain in 14 villages.

**Methods:**

A cross‐sectional study was conducted in Lushoto and Handeni districts whereby 93 randomly selected respondents constituting farmers, vendors and restaurants/kiosks were interviewed and subsequently 166 milk samples were collected for microbiological enumeration and ascertain contaminations by *Escherichia coli* O157:H7 and *Brucella abortus* using standard protocols. Chi‐square (*χ* ^2^) and confidence intervals were used to compare proportions of risk factors for milk microbial contaminations; a probability of *p* < 0.05 was statistically significant. Logistic regression was used to assess the significant risk factors associated with milk microbial contaminations.

**Results:**

It was established that milking and milk storage was done under unhygienic conditions. Up to 92.1% of farmers had no training on livestock husbandry and milk handling practices. Mean bacterial counts in milk ranged between 3.3 and 5.8 log_10_, which is in line with the East African Standards (5.3 log10 cfu/mL). However, 87% and 93% of milk from farmers and vendors, respectively, had bacteria count above the recommended EAC standards of 2.0 × 10^5^ cfu/mL implying poor microbiological quality. All milk samples had coliform plate count above EAC standards of 4.5 log CFU/mL. The mean bacterial count of 5.3 log10 cfu/mL was recorded with more counts in milk from vendors (4.6 to 6.1 log10 cfu/mL). Average levels of contamination in raw and pasteurised milk were not significantly different (*p* > 0.05). Factors related to hygienic practices of farmers along the milk production chain had no significant differences (*p* > 0.05) in microbial contamination. There was no *E. coli* O157:H7 contamination in milk, nonetheless *B. abortus* was detected in 37 out of 87 samples tested (42.5%) mostly from farmers.

**Conclusions:**

Limited extension services, unhygienic practices along milk value chain contributed to high microbial contamination. Detection of *B. abortus* in milk is of public health significance due to its zoonotic potential. Therefore, the government and milk stakeholders should strengthen public and private livestock extension services for proper animal husbandry practices, improved milk value chain and control of zoonotic diseases.

## Graphical abstract

Milk and milk products are important sources of nutrients to human and animals, but due to its physicochemical characteristics and nutritional value, it serves as an excellent medium for growth of microorganisms. Most of smallholder dairy farmers have no training on livestock husbandry and milk handling practices. The cow milk always has high load of bacterial contamination above East African Standards (5.3 log10 cfu/mL). Zoonotic bacteria like *Brucella abortus* contamination rate are at 42.5%. Poor livestock husbandry practices coupled with unhygienic practices along milk value chain account for all these problems which call for urgent need for interventions for prosperity of dairy industry and safeguard public health.

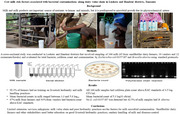

## 1 Introduction

Food‐borne diseases are a serious threat to people in Africa, responsible for 33%–90% cases of mortality in children (Flint et al. 2005). Although foods of animal source are a minor constituent in most diets, they are responsible for majority of incidents of food‐borne illness (De Buyser et al. 2001; Ngasala et al. 2015; Gwandu et al. 2018). Despite being a nutritional‐balanced foodstuff, milk serves as a medium that favours growth of several microorganisms. Up to 90% of all dairy related diseases are due to pathogenic bacteria found in milk (Weinhäupl et al. 2000; Crump et al. 2013; Cash‐Goldwasser et al. 2018; Anga et al. 2024). Studies on milk microbial contaminations report on several zoonotic bacteria, namely, *Brucella* sp., *Mycobacterium bovis*, *Coxiella burnetii*, *Leptospira* sp., *Salmonella* sp., *Escherichia coli*, *Listeria monocytogenes* and *Campylobacter* (Katale et al. 2012; Katale et al. 2013; Msalya 2017; Ukita et al. 2021; Mpatswenumugabo et al. 2023; Mengele et al. 2023). Other common bacterial contaminants are *Staphylococcus* spp., *Streptococcus* spp., *Actinobacter pyogenes, Pseudomonas aeruginosa*, *Proteus* spp., *Bacillus cereus, Yersinia enterocolitica*, *Clostridium perfringens* and *Corynebacterium* spp. (Abdel‐Rady and Sayed 2009; Hosseinzadeh and Saei 2014; Msalya 2017; Ashraf and Imran 2020). Bacteria have potential for causing milk‐borne diseases to humans. Bacteria may get access to milk from the primary source shed from the animal with infections or secondary contamination along the value chain (Ngasala et al. 2015; Gwandu et al. 2018; Mengele et al. 2023; Mpatswenumugabo et al. 2023). In recent years there have been emergency of new pathogenic bacteria along the food chain such as *Eschericia coli* 0157:H7 (Sivapalasingams et al. 2004; Schoder et al. 2013; Lupindu et al. 2014). Unlike in developed countries, the dairy industry in most African countries is underdeveloped, dominated by unpasteurised milk and informal markets that gives all possibilities for contaminations (Ngasala et al. 2015; Gwandu et al. 2018; Lugamara et al. 2023).

Raw milk is a major means for transmission of food‐borne pathogens to humans. The annual milk production in Tanzania is almost 4 billion litres and the production is expected to keep on increasing due to the increased demand for milk (MLF 2023). However, more than 90% of the milk produced in Tanzania is consumed in unprocessed form because of the produced milk is sold in informal market operated by smallholder producers. The milk market faces several constraints including high risks of biological, chemical and physical contaminations; and high postharvest losses due to spoilage (Lugamara et al. 2023; Mengele et al. 2023; Mashauri et al. 2025; Makoye and Nonga 2026). Nonetheless, there are several legislations regulating milk as food, which include The Animal Diseases Act Cap 156 (2003), The Dairy Industry Act Cap 262 (2006), The Standards Act Cap 130 (2009), The Public Health Act, Cap 263 (2009) and The Tanzania Food and Nutrition Act CAP 109 (2009). The problem has been enforcing laws to ensure safety of the milk along the value chain.

In Tanzania, the milk value chain considers the informal and formal milk markets all together. It is constituted of five main segments namely farmers or producers, milk collection centres, processors, transporters or distributors, and sellers or exporters (Mbiha 2008; Ogutu et al. 2014; Katjiuongua and Nelgen 2014). The informal market channel dominates the chain by 90% (TLMP 2017; Mbiha 2008; Katjiuongua and Nelgen 2014; Lugamara et al. 2023; Lunogelo et al. 2021; Eshitera et al. 2023). The informal chain mainly constitutes the consumers of unprocessed that include the farmers and their neighbours, food vendors, hotels, kiosks, groceries, coffee centres, restaurants, schools, public offices and other public places. The follow up and regulation of this is channel is not easy since it carries most of the actors and normally under informal set up. By large, apart from home consumption, farmers may sell milk to neighbours, hawkers and collectors or traders who in turn sell to consumers (Mbiha 2008; Ogutu et al. 2014; Katjiuongua and Nelgen 2014; Lunogelo et al. 2021). The informal channel suffers from number of challenges including poor‐quality milk handling equipment, microbial, chemical or physical contaminations and adulterations, and limited or absence of cold chain (Mbiha 2008; Ogutu et al. 2014; Katjiuongua and Nelgen 2014; Ngasala et al. 2015). Economically, more than 25% of the milk in the chain is spoiled and condemned that causes losses to farmers and other players in the chain (Lugamara et al. 2023, MLF 2023; Mbiha 2008). Further, the chain is documented to significantly contribute to milk‐borne diseases and other public health problems. Despite all these shortfalls in this type of channel, unfortunately, it serves majority of the milk consumers in Tanzania and elsewhere in Africa (Ogutu et al. 2014; Ngasala et al. 2015; Eshitera et al. 2023). Indeed, farmers and milk hawkers prefer the channel due to lack of rigorous milk quality inspection, limited availability of milk collection centres and milk processing factory, poor quality of milk, good prices of milk, prompt payments, readily availability of customers and assumed good milk prices (Massawe et al. 2018; Mbiha 2008; TLMP 2017; Lugamara et al. 2023).

The formal milk chain involves the milk collection centres owned and operated by the processing companies and cooperatives (Mbiha 2008; Ogutu et al. 2014; Katjiuongua and Nelgen 2014). The number of milk collection centres are more than 260 which collect more than 95 million litres of milk per year (MLF 2023). The MCC transport milk to processing plants which make different dairy products including liquid milk products like pasteurised, homogenised, UHT, fortified, and skim milk; cream products like cream; butter and fats fermented dairy like yogurt and sour milk; cheese; concentrated or dried products like powdered milk and frozen desserts like ice cream. The dairy products mostly are locally marketed mainly in shops, supermarkets, restaurants and hotels and average of 130,000 litres is exported (Mbiha 2008; Ogutu et al. 2014; Katjiuongua and Nelgen 2014; MLF 2023). The formal milk chain at least has better cold chain and involves rigorous milk inspection and tests at milk collection points or centres and processing plant. The tests include sensory or organoleptic tests like smell, colour, debris or dirt, alcohol or clot on boiling, milk density by lactometer test, iodine test for starch adulteration, California mastitis tests (CMT), somatic cell count, physicochemical assessment like butter fats and other milk solids (Mbiha 2008; Ogutu et al. 2014; Katjiuongua and Nelgen 2014; Gwandu et al. 2018). Levels of bacterial load are measured by Resazurin test and occasionally antimicrobial residue test (The Dairy Industry Act Cap 262 2006; UNDP/BCS/TetraPak 2006; Mbiha 2008; The Standards Act Cap 130 2009; Gwandu et al. 2018; Eshitera et al. 2023).

Presence of milk collection centres interconnected with large‐scale milk processing plants in Tanga region Tanzania accelerated many smallholder dairy farmers in Handeni and Lushoto districts to increase the milk production because of assured market. Meanwhile information on milk handling, risks associated with informal market and unhygienic handling of milk from producer level to consumer is limited. Therefore, this study was conducted to assess the milk handling practices, bacterial contaminations and determine selected milk‐borne zoonotic pathogens along the dairy value chain in Lushoto and Handeni districts in Tanga region.

## 2 Materials and Methods

### 2.1 Description of the Study Area

The study was conducted in Tanga region of the northeastern coastal part of Tanzania. The area is located between longitudes 37° and 39°East and latitudes 4° and 6°South and is characterised by hot and humid tropical climate with rain seasons in March, April, November and December. The mean annual rainfall varies from 500 to 1400 mm with relative humidity ranging from 60% to 90% for most of the year.

**FIGURE 1:**
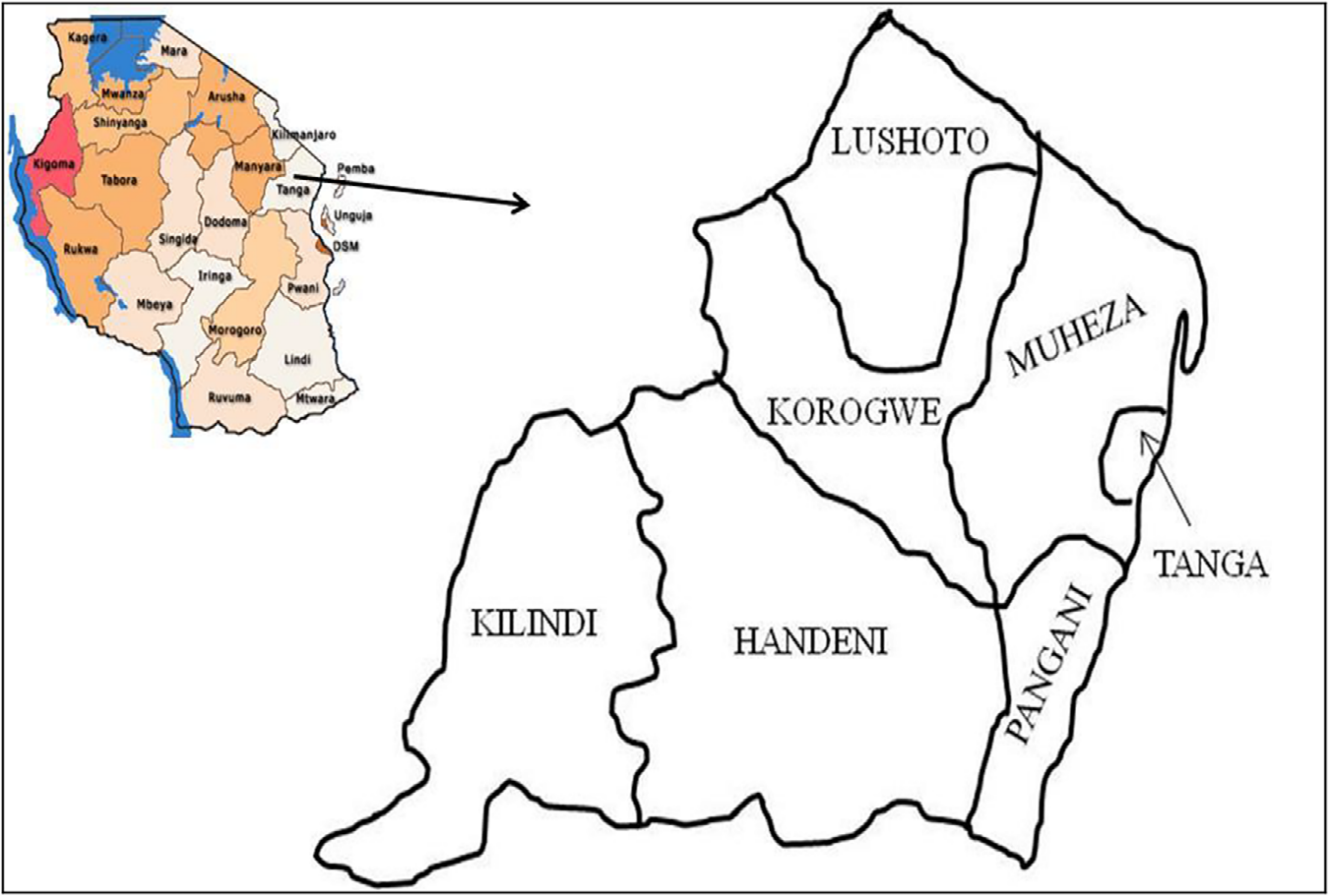
A map of Tanga region showing its districts including Lushoto and Handeni districts that were selected in this study: *Insert* is a map of Tanzania that shows different regions.

Tanga region was purposively chosen for the study due to its long history of livestock keeping and dairy marketing owing to support by several Non‐Governmental Organizations of Smallholder Dairy Development programmes. The region also has large‐scale milk processing plants interconnected with milk collection centres which support small scale dairy production. Lushoto and Handeni are among the are among the district Councils with high number of smallholder dairy farmers in the region. The location of the study districts Lushoto and Handeni are shown in Figure 1.

### 2.2 Study Design, Sample Size and Population

A cross‐sectional study design was employed. The sample size of 184 milk was calculated as described by Kothari (2004), however only 166 samples from 93 participants (65 smallholder dairy farmers, 16 vendors and 12 operators of restaurants or kiosks) were collected and analysed. The reasons for reduced sample handled included dry season where most households keeping cattle in the study villages had no lactating cows and unwillingness to participate in the study. The smallholder dairy farmers fed their animals through zero grazing and kept crossbreeds of dairy cattle, and traditional farmers extensively grazed their animals, and they kept mainly local breeds, namely, Tanzania shorthorn Zebu.

The study districts were purposely selected by Safe Food, Fair Food (SFFF II) project based on criteria of number of livestock keepers and accessibility. With the help of Ward Livestock Field Officers (LFO), households with lactating cows in the selected 14 study villages were identified and prepared a list in each village that was used as sampling frame. The smallholder farmers in the specific 14 village were obtained by simple random sampling. The district livestock officer and Ward Livestock Field Officers (LFO) guided the process of obtaining the other players in the milk value chain. The milk retailers studied were purposely selected in each district categorised as milk vendors and restaurants or kiosks. The inclusion criteria of the study participants included availability of milk during the study, accessibility and willingness to participate in the research. A total of 65 farmers and 28 milk retailers were selected.

### 2.3 Questionnaire Administration

Administration of structured questionnaires to respondents was done before sampling milk. During administration of questionnaires, direct observation on general cleanliness and hygienic practices in the milk value chain was done. The questionnaires to farmers collected information on livestock husbandry practices, diseases challenges, milk production, milking and milk handling, marketing and selling, transportation and common problems associated with milk. For the retailers, the questionnaire aimed at getting information on the quality of milk, possible sources of microbial contamination and problems associated with milk trading. Prior to start of data collection, the questionnaires were pretested and revised accordingly. The revised questionnaires were translated into Kiswahili, the language known by the respondents.

### 2.4 Milk Sample Collection and Transportation

After questionnaire, 50 mL of milk sample was collected from all the actors in the dairy value chain. The samples were obtained from the milk containers, put in a sterile falcon tube, and placed in a cool box with ice packs and transported to Lushoto and Handeni districts office where were initially stored at –20°C. Subsequently, the samples were transported to the Sokoine University of Agriculture (SUA) laboratories and analysed for bacteria contamination. A total of 166 (108 raw and 58 pasteurised) milk samples were collected. Of the raw milk, 80 samples were collected from the farmers and 28 from vendors. The 58 pasteurised milk samples were sampled from restaurants/kiosks.

### 2.5 Microbiological Analysis of Milk

All the 166 milk samples were analysed for total bacteria count (TBC) and total coliform count (TCC). Different media (Oxoid Ltd., Basingstoke, Hampshire, England, UK) were prepared according to manufacturer's instructions and stored at 8°C refrigeration until use. Total bacteria count in milk at 37°C was done using a protocol described by TZS (2007) and ISO/FDIS (2001). Microbial colon count on culture media was done as described by ISO 7218 (2007). Briefly, tenfold serial dilution of milk sample from 10^−1^ to 10^−10^ in sterile normal saline solution was done. From each dilution, 1 mL of diluted sample was placed in a sterile Petri dish followed by the addition of 20 mL of molten nutrient agar (Oxoid Ltd., Basingstoke, UK), gently shaken, and left to solidify. Incubation was done under the aerobic condition at 37 ± 1°C for 24 ± 3 h. The microbial colon count on the plates was done with the aid of portable magnifying lens and colonies in the culture plate were countered by using colony counter. Only the plates with 30–300 colonies were considered in calculating the colony forming units (CFU) per mL of sample. According to EAC (2006) standards, the TBC should not exceed 5.3 log colony forming units per millilitre (cfu/mL) of raw cow milk.

For total coliform count (TCC), the raw milk samples were thoroughly shaken and diluted as for TBC. The samples were inoculated on MacConkey's agar (Oxoid Ltd., Basingstoke, UK) using the spread plate method. The plates were then incubated at 37°C for 36 h (Harley 2013). The number of colonies was recorded using a colony counter. Only the plates with 30–300 colonies were considered in calculating the colony forming units per mL of sample. TCC exceeding 4.5 log CFU/mL was grouped as above recommended levels (EAC 2006). The number of counted bacteria was expressed in colony forming units per mL using the following formula:

$$\mathrm{Number}\;\mathrm{of}\;\mathrm{bacteria}=\frac{\mathrm{Number}\;\mathrm{of}\;\;\mathrm{colony}\;\mathrm{forming}\;\mathrm{unit}\left(\mathrm{cfu}\right)}{\mathrm{Volume}\;\mathrm{plated}\left(\mathrm{mL}\right)\times \;\mathrm{total}\;\mathrm{dilution}\;\mathrm{factor}}$$

### 2.6 Molecular Analysis of Milk Bacterial Contaminants

A total of 166 milk samples underwent testing for *E. coli* O157:H7 contamination, while 87 samples were examined for *B. arbutus* infection. Conventional PCR was used to identify the two bacteria in milk samples using their specific primers. A GeneAmp 9700 thermal cycler (Applied Biosystems, Foster City, CA) was used for the amplification process. A UV Transilluminator (VWR International BVBA, Leuven, Belgium) was used to view the PCR results. The milk sample was boiled at a temperature of 80°C for 30 min, followed by centrifugation at 17,000 × *g* for 5 min. The pellet was discarded and supernatant used for DNA extraction. DNA extraction was done from the supernatant using the ‘QIAamp Viral Mini Kit‐Qiagen’ (QIAGEN Sciences, Maryland, USA) as per the manufacturer's instructions. In house *E. coli* O157:H7 and *B. abortus* isolates were used as positive controls. DNA extraction was done by boiling the isolates at 80°C for 30 min in a thermo‐cycler followed by centrifugation at 17,000  × *g* for 5 min. The pellet was discarded and supernatant taken.

PCR primers BRU‐ P5 and BRU‐ P8 were used for the PCR amplification of the rDNA of *B. abortus* while primers O157‐3 and O157‐4 were used for the PCR amplification of the hyl A gene of *E. coli* O157:H7. The primer sequences as indicated in Table 1.

**TABLE 1:** Primer sequences used for *B. abortus and E. coli* O157:H7.

Organism	Primer name: primer sequence (5’‐3’)	Target gene	Reference
*B. abortus*	BRU‐P5: TCGAGAATTGGAAAGAGGTC BRU‐P8: GCATAATGCGGCTTTAAGA	16S‐23S 16S‐23S	Nancy et al. (1996)
*E. coli*	0157‐3: GTAGGGAAGCGAACAGAG 0157‐4: AAGCTCCGTGTGCCTGAA	*hly* A *hyl* A	Wang et al. (1997)

The *B. abortus* 16s‐23s sequence was amplified as previously described by Nancy et al. (1996) with some modifications in the total master mix and annealing temperature. Briefly, PCR was performed in a total volume of 25 µL containing 0.5 µL of *Taq* DNA polymerase, (Invitrogen) 12.5 µL of 2× reaction buffer, 7 µL of RNase free water, 10 pmol of each primer and 3 µL of DNA template. The mixture was subjected to 40 cycles of amplification in a thermal cycler (StepOne PCR systems, Applied BioAsystems). The initial denaturation was for 10 min at 95°C. The cycle consisted of denaturation for 30 s at 95°C, annealing for 30 s at 55°C and extension at 72°C for 30 s. The final extension step was performed at 72°C for 10 min.

The *E. coli hyl*A gene was amplified as previously described by Wang et al. (1997) with some modifications. Briefly, the PCR mixture consisted of 25 µL containing 0.5 µL of *Taq* DNA polymerase (Invitrogen), 12.5 µL of 2× reaction buffer, 8 µL of RNase free water, 10 pmol of each primer and 2 µL of DNA template. The PCR reaction was performed in a thermo cycler at a denaturation temperature of 72°C for 10 min. A total of 35 cycles at 95°C, 55°C and 72°C each for 30 s followed denaturation. The final extension was performed at 72°C for 10 min. After DNA amplification, PCR products were analysed using 1.5% agarose gel at 100 Voltages for 30 min and visualised under and ultraviolet trans‐illuminator.

A volume of 5 µL of the PCR products was mixed thoroughly with 1 µL of blue/orange 6× loading dye (Promega—Madison USA) and were loaded in agarose gel wells. Further, 10 µL of a 1 kb molecular weight marker mix (Promega—Madison USA) was loaded in a parallel track. The horizontal gel electrophoresis was carried out at a constant voltage of 100 V for 30 min.

### 2.7 Data Analysis

The data collected was entered into Microsoft office excel worksheet for cleaning and preliminary analysis. The cleaned data was then copied into STATA I/C 11 statistical package for further analysis. Descriptive statistics like mean, frequencies and percentages were extracted and data presented accordingly. Chi‐square (*χ* ^2^) and confidence intervals were used to compare proportions of categorical variables like risk factors for milk microbial contaminations. Relationship between different practices as risk factors for microbial contamination in milk was computed against TPC and TCC and statistical significance was established at 95% confidence and critical *p* value of 0.05. Logistic regression was used to assess the significant risk factors associated with milk microbial contaminations.

## 3 Results

### 3.1 Demographic Characteristics of the Respondents

A total of 93 participants (65 farmers, 28 retailers) were interviewed. The results showed that there were more male farmers and milk vendors; while more females were realised in restaurants/kiosks. A total of 14 villages were included in the study (Table 2).

**TABLE 2:** Demographic characteristics of respondents.

		Number of respondents (%)			
Demographic information	Category	Farmers*n* = 65	Vendors*n* = 16	Restaurants/kiosks*n* = 12	Total*n* = 93
Gender	Male	49 (75.4)	11 (68.8)	5 (41.7)	65 (69.9)
	Female	16 (24.6)	5 (31.3)	7 (58.3)	28 (30.1)
Districts	Lushoto	36 (55.3)	0 (0.0)	8 (66.7)	44 (47.3)
	Handeni	29 (44.6)	16 (100)	4 (33.3)	49 (52.7)
Number of respondents from each village	Ubiri	10 (15.8)	0 (0.0)	2 (16.7)	12 (19.9)
	Magamba	10 (15.3)	0 (0.0)	1 (8.3)	11 (11.8)
	Chakechake	6 (9.2)	0 (0.0)	2 (16.7)	8 (8.6)
	Irente	2 (3.1)	0 (0.0)	0 (0.0)	2 (2.2)
	Hamboyo/viti	8 (12.3)	0 (0.0)	3 (25)	8 (8.6)
	Konji	9 (13.8)	4 (25)	1 (8.3)	14 (15.1)
	Kwediyambu	7 (10.7)	1 (6.3)	1 (8.3)	9 (9.7)
	Sindeni	4 (6.1)	5 (31.3)	1 (8.3)	10 (10.6)
	Chanika	6 (9.2)	0 (0.0)	0 (0.0)	6 (6.6)
	Kibaya	3 (4.6)	0 (0.0)	0 (0.0)	3 (3.2)
	Kilimila	0 (0.0)	1 (6.2)	0 (0.0)	1 (1.1)
	Kolanda	0 (0.0)	2 (12.5)	0 (0.0)	2 (2.2)
	Kwemsiha	0 (0.0)	1 (6.2)	0 (0.0)	1 (1.1)
	Malezi	0 (0.0)	2 (12.5)	0 (0.0)	2 (2.2)

### 3.2 Management and Health Issues of Cattle

Table 3 shows the cattle husbandry practices and health in the study areas. Majority of the study households in Handeni kept indigenous Zebu cattle that were under extensive grazing system different from Lushoto where most farmers had crossbred of dairy cattle preferably Friesian. Most of the cattle houses were made of trees, and earthen floors without a roof that allowed water to log during rainy season. It was realised that majority of farmers (92.1%) had no training on good livestock husbandry practices and general issues related to milk and dairy products. On animal health issues, only 23.1% reported to routinely vaccinate their animals. Several disease problems were reported including tick‐borne diseases. Most farmers treated their animals, and they sourced the veterinary drugs from veterinary shops and livestock markets.

**TABLE 3:** Cattle management practices and health in the study farmers (*n* = 65).

Variable	Category	No. (%) respondents
Breed of cattle	Indigenous breed (Zebu)	28 (43.1)
	Crosses of dairy breed	37 (56.9)
Type of feeding	Extensive grazing	33 (50.7)
	Zero grazing	32 (49.3)
Use of supplementary feed	Maize bran	6 (9.5)
	Mineral supplement	6 (9.5)
	Others	7 (11.1)
	No supplementary feed to cattle	46 (70.8)
Types of animal house	Boma made of tree	46 (70.8)
	Tree slabs and bricks	19 (29.2)
Animal house floor materials	Mud/earthen	58 (89.2)
	Concrete/cement	7 (10.8)
Animal house floor cleaning	Yes	36 (55.4)
	No	29 (44.6)
Routine cleaning of animal house floor	Once a day	16 (24.6)
	Twice a day	7 (10.8)
	Once a week	12 (18.5)
	Others	30 (46.1)
Training in good livestock husbandry and milk handling practices	Yes	5 (7.8)
	No	60 (92.2)
Routine vaccination of cattle	Yes	15 (23.1)
	No	40 (76.9)
Routine use of acaricides	Yes	30 (46.2)
	No	35 (53.8)
Routine screening of diseases of cattle	Yes	0 (0.0)
	No	65 (100.0)
Disease problems	Tick‐borne diseases like ECF	34 (52.3)
	Trypanosomiasis	15 (23.1)
	Emaciation thought to be bovine tuberculosis	2 (3.1)
	Anthrax	1 (1.5)
	Not reported	13 (20.5)
Who treats sick cattle	Farmer themselves	39 (60.0)
	Livestock extension officers	26 (40.0)
Medicines for treatment of cattle	Herbs	34 (52.3)
	Veterinary drugs	31 (47.7)
Sources of veterinary drugs	Veterinary shops	30 (46.2)
	Other sources like livestock markets	35 (53.8)

### 3.3 Hygienic Practices Along Milk Supply Chain

The main source of water for use in milk supply chain was tap water (40%). The common type of containers used during milking, storage and distribution were the wide and narrow necked plastic containers. There were no cold storage facilities along the supply chain. Majority of the farmers washed milking utensils, hands, cow teats and cleaned animal sheds before milking using cold water with no detergent. All the farmers reported to do hand milking and on observation, the milking places were always dirty. The most common means of transport used by farmers in delivering milk was on foot (Table 4).

**TABLE 4:** General practices during milking, storage and delivery of milk (*n* = 65).

Variable	Category	No. (%) farmers respondents
Sources of water	Tap	26 (40.0)
	Wells	21 (32.3)
	Dams and/or streams	18 (27.7)
Milking practices	Hand milking	65 (100.0)
	Cleaning animal shed before milking	28 (43.1)
	Wash milking utensils before milking	60 (92.3)
	Wash hands before and after milking	47 (72.3)
	Wash cow's teats before milking	41 (63.1)
Containers used for milk storage	Wide necked aluminium vessel	2 (3.1)
	Wide necked plastic vessel	56 (86.1)
	Used water and oil bottles	6 (9.2)
	Cooking pan ‘sufuria’	1 (1.5)
Containers used for delivery/transportation	Wide necked aluminium vessel	0 (0.0)
	Wide necked plastic vessel	38 (58.5)
	Used water and oil bottles	8 (12.3)
	Cooking pan ‘sufuria’	3 (4.6)
	Others e.g. traditional pots	16 (24.6)
Means of delivery	On foot	37 (56.9)
	By bicycle	9 (13.8)
	By motorcycle	3 (4.6)

### 3.4 Practices by Retailers in Sale and Storage of Milk

Most of the milk retailers reported buying milk from farmers in their villages where they bulk and sell. The retailers sold the milk to individuals, collection centres and restaurants/kiosks. Milk was mostly sold and/or distributed in plastic containers. Additionally, the milk sellers received no training on proper handling techniques or milk quality. Majority of the vendors and restaurants/kiosks have experienced losses due to spoiled milk (Table 5).

**TABLE 5:** Source, sale and storage of milk by retailers.

Variable	Category	Number of respondents (%)	
		Vendors	Restaurants/kiosks
		*n* = 16	*n* = 12
Source of milk	A specific farmer in the same village	3 (18.7)	0 (0.0)
	More than one farmer in the same village	11 (68.7)	5 (41.7)
	Farmers in the nearby village	2 (12.5)	3 (25)
	Vendor from the same village	0 (0.0)	3 (25)
	Milk collection centres	0 (0.0)	1 (8.3)
Type of milk sold	Raw milk	11 (68.7)	1 (8.3)
	Boiled milk	5 (31.3)	11 (91.7)
Milk customers	Neighbouring households	6 (37.5)	—
	Collection centres	7 (43.7)	—
	Passersby	3 (18.7)	—
Containers used for milk delivery/selling	Wide necked plastic vessels	10 (62.5)	10 (83.3)
	Narrow necked plastic vessel	4 (25.0)	2 (16.7)
	Traditional pots	2 (12.5)	0 (0.0)
How milk is served	Cup	2 (40.0)	—
	Soda/water bottles	3 (60.0)	—
	Hot from a thermal flask in a cup	0 (0.0)	9 (75.0)
	Hot from a cooking pan in a cup	0 (0.0)	3 (25.0)
Handling/storage of excess milk	Consume	—	8 (66.7)
	In a fridge	—	1 (8.3)
	Room temperature; re‐boil next day for sale	—	3 (25.0)
Ever encountered challenges of spoiled milk	Yes	13 (81.3)	9 (75.0)
	No	3 (18.7)	3 (25.0)

### 3.5 Total Bacteria Count and Total Coliform Count

The results of TBC and TCC for milk from farmers, from vendors and from restaurants are summarised in Table 6. The results showed a mean TBC of 5.3 log10 cfu/mL with more counts recorded in milk from vendors ranging from 4.6 to 6.1 log10 cfu/mL. According to the East African Community standards of raw cow milk (EAC 67:2007), a good quality raw cow milk should have TBC of less than 5.3 log10 cfu/mL. The results showed that 87.5% of milk from famers and 92.9% of milk from vendors had TBC above the EAC recommended level of 2.0 × 10^5^ cfu/mL. This implied that most milk from farmers and vendors had poor microbiological quality.

**TABLE 6:** Total bacteria counts and total coliform count for milk samples from the actors in the value chain.

Milk source	Number of samples	Mean (log_10_ cfu/mL)	SD (log10)	Min	Max
TBC					
Farmers	80	5.3	5.4	3.3	5.8
Vendors	28	5.8	5.7	4.6	6.1
Restaurants	58	4.9	4.9	0	5.3
TCC					
Farmers	80	4.8	4.9	2.5	5.5
Vendors	28	4.8	5.1	3.3	5.4
Restaurants	58	3.6	3.9	0	4.3

The mean TCC was found to be 4.3 (log10 cfu/mL) with more counts recorded in vendors which ranged from 3.3 to 5.4 (log10 cfu/mL) as indicated in Table 6. Meanwhile according to East African community standards for CPC of raw milk (EAS 67:200), good quality raw cow milk should not exceed TCC of 3 (log10 cfu/mL). This implied that all the milk samples analysed for CPC were above the recommended EAC levels for TCC. In reference to this limit, it indicates unhygienic handling of milk. The average levels of contamination in raw and pasteurised milk (5.3 log10 cfu/mL and 4.9 log10 cfu/mL, respectively, for TBC, and 4.3 log10 cfu/mL and 3.6 log10 cfu/mL, respectively, for TCC) were not significantly different (*p* > 0.05).

### 3.6 Risk Factors for Microbial Contamination of Milk at Farmers’ Level

Several factors related to hygienic practices of the farmers during milking, handling and storage of milk were possible risk factors for microbial contamination as reflected by TBC and TCC. The factors considered in the analysis included unhygienic hands, cow teats, animal houses and types of milk containers. Statistically, all the factors were found to be not significant (*p* > 0.05) causes of high TPC and CPC (Table 7). However, Table 8 shows an increased trend of microbial counts in relation to washing hands and cleaning animal house before milking. The microbial counts increased when actors either did not wash hands or cleaned animal houses.

**TABLE 7:** Possible risk factors associated with microbial contamination of milk at farmers’ level, *p*‐value at 95% CI.

Milking and milk containers	Risk factors	Per cent	*p*‐value	Mean TPC	Mean CPC	*p*‐value
Milking practices before milking	Wash hands	72.3	0.47	2 × 10^5^	5.9 × 10^4^	0.48
	Wash cow teats	63.1	0.52			0.40
	Clean animal house	43.1	0.26			0.31
Types of containers	Wide necked aluminium container	13.6				
	wide necked plastic container	72.7	0.35	2 × 10^5^	5.9 × 10^4^	0.39
	Cooking pan ‘sufuria’	13.6				

**TABLE 8:** Bacteria count in milk in relation to hygienic practices.

Total plate count	Wash hands before milking	Wash cow teats before milking
2180	Yes	Yes
2700	Yes	Yes
3550	Yes	Yes
4065	Yes	Yes
8850	Yes	No
18,400	Yes	Yes
21,520	Yes	Yes
31,900	Yes	Yes
31,900	Yes	Yes
42,000	Yes	Yes
56,700	Yes	Yes
120,500	No	Yes
125,000	No	Yes
190,000	No	Yes
478,000	No	No
548,000	No	No
567,700	Yes	No
666,000	No	No

### 3.7 Risk Factors for Microbial Contamination of Milk at Vendors and Restaurants/Kiosks’ Level

Risk factors that were associated with the microbial contamination of milk from vendors and restaurants included sources of milk, type of containers used for delivery and serving and/or storage of milk, means of transport during delivery and preparation of milk for selling. However, all these factors when analysed against TPC and CPC were found to be not statistically significant (*p* > 0.05) (Table 9).

**TABLE 9:** Risk factors associated with milk containers for milk vendors and restaurants.

Factors assessed	Category	Vendors milk percentage	Restaurants/kiosks milk percentage	*p*‐value (TPC)	*p*‐value (CPC)
Sources of milk	One farmer		20.0	0.28	0.116
	> One farmer		80.0		
Type of milk	Raw	60.0		0.128	0.261
	Boiled	20.0			
Containers for selling milk	Wide necked aluminium container	57.1		0.32	0.42
	Wide necked plastic container	42.9			
Milk delivery to customers	From stationary selling point		85.7	0.32	0.71
	Moving restaurant/kiosk		14.3		
Container used for selling	Narrow necked plastic container	80.0		0.28	0.26
	Wide necked plastic containers	20.0			
Vendors transportation of milk to customers	By bicycle	60.0		0.27	0.23
	By motorcycle	40.0			

### 3.8 PCR Determination of *E. coli O157:H7* and *B. abortus*

**FIGURE 2:**
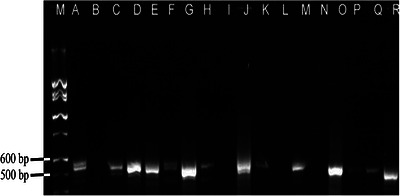
Detection of *B. abortus* by PCR using BRU P5 and BRU P8 primer pairs targeting 16S‐23S gene at between 500 to 600 bp. Note that lane M is a molecular weight marker while lanes A, C, D, E, F, G, H, J, K, M, O, P and Q are positive amplicons whereas lane B, I, L and N are negative amplicons. R is positive control.

The molecular results showed that all the 166 milk samples tested were negative for *E. coli* O157:H7. However, of the 87 milk samples tested for *B. abortus* infection, 37 (42.5%) were positive majority (28.7%) of which were sourced from farmers (Table 10, Figure 2).

**TABLE 10:** *B. abortus* PCR results for milk samples from Handeni and Lushoto.

Source of milk samples	Lushoto (%) *n* = 45	Handeni (%) *n* = 42	Total (%) *n* =87
Restaurant	3 (6.7)	6 (14.28)	9 (10.3)
Farmers	14 (31.1)	11 (26.2)	25 (28.7)
Vendors	0 (0.0)	3 (7.1)	3 (3.5)
Total	17 (37.8)	20 (47.6)	37 (42.5)

## 4 Discussion

This study aimed to assess the milk handling practices and bacterial contaminations along the dairy value chain in Lushoto and Handeni districts of Tanga region. Possible risk factors for microbial contaminations along the dairy value chain were explored and the involvement of *B. abortus* and *E. coli* O157:H7 as important milk‐borne pathogens was elucidated by using polymerase reaction. This was because most of the milk used in Tanzania comes from informal sector, which is rarely regulated by the existing legislations. It has been established that the general animal housing and feeding, animal health and management, practices of milk harvesting, storage, transportation and retailing predisposed the milk to microbial contamination. Bacteriologically, high TPC and CPC were encountered in most of the samples, which were above the recommended East Africa Community standards (EACs 2006). Interestingly, high prevalence of *B. abortus* was recorded in milk, which endangers the health of the milk consumers. Fortunately, *E. coli* 0157:H7 was not detected in all the milk samples analysed. These results call for continuous education for the smallholder farmers and other players on good livestock husbandry practices, control of animal diseases and hygienic handling practices of milk along the value chain to safeguard public health and avoid losses due to milk spoilage.

The study established that cattle farming in Handeni district is dominated by smallholder farmers that keep indigenous short horn Zebu that are fed under extensive grazing system different for Lushoto most farmers, most farmers keep crossbred of dairy cattle which some are fed under semi‐intensive system and others under zero grazing. The type of livestock production system determined the level of husbandry practices. Majority of the indigenous cattle were housed in an open roof house made of trees, earthen floor that had manure accumulated on the floor and allowed water logging during rainy season leading to a muddy nasty environment. This set up predisposes cattle to different health problems that include foot rot, mastitis and skin diseases (Aiello and Moses 2006). Nonetheless, crossbreed dairy cattle were relatively better managed in a house with a wall made of tree slabs and bricks, with a roof and a concrete or cemented rough floor that permits cleaning. Crossbreed dairy cattle were fed under zero grazing while others under semi‐intensive with some concentrate supplementation. This was a relatively better type of husbandry that can minimise health problems and improve production. The study also found that none of the 65 farmers’ studies had ever got some training on good husbandry practices and general issues related to milk and dairy products. This accounts for any shortfall observed in the livestock husbandry practices and unhygienic practices in milk harvesting, handling and storage.

Health status of the cattle was not appealing since apart from poor livestock management, there were minimal or no routine animal diseases control programs on the farms. Only 23.1% of farmers vaccinated their cattle, acaricide use was at 46.2% and biosecurity measures none existed. This situation probably facilitated occurrences of livestock diseases like tick‐borne diseases in particular East coast fever, trypanosomiasis and poor health condition, which was mentioned as Bovine tuberculosis and anthrax. The cattle health challenges necessitated farmers to source veterinary drugs from agrovet shops and livestock markets and treat their animals themselves. According to Animal diseases Act Cap 156 (2003), vaccination against priority diseases and acaricide application is mandatory. Further, The Veterinary Act Cap 319 (2003) and The Tanzania Food, Drugs, and Cosmetics Act Cap 219 (2003) restrict haphazard sales and use of veterinary drugs, but the problem has been on enforcement especially in rural areas. Livestock farmers in Tanzania have free access to any veterinary drugs over the counter and open livestock market without any prescriptions which fuels the indiscriminate uses and consequently leads to antimicrobial residues, antimicrobial resistance and other health effects (Katakweba et al. 2012; Mdegela et al. 2021; Sangeda et al. 2021). Therefore, livestock extension services should be available to farmers to foster good livestock husbandry practices and appropriate animal diseases control measures. This should go along with enforcement of laws to curb uncontrolled sales of veterinary drugs is of paramount important. This may help to possibly eradicate the problem of antimicrobial residues in food of animal origin.

Microbial contamination of raw fresh milk may originate from the cattle or secondary sources after milking. Mastitic udder sheds high number of bacteria into the milk. The poor hygiene of udder, milking utensils, parlour and the personnel substantially add bacteria to the milk. It is recommended that before milking, there should be thorough sanitation of the udder, milking environment, equipment and personnel. Teat dipping in suitable disinfectants is necessary to control entry of microorganisms through the teat canal. During this study, majority of farmers harvested, stored and distributed milk under unhygienic conditions using milk containers made of plastic, which predisposed milk to secondary microbial contamination. Worse enough, storage and handling milk under room temperature increases bacteria multiplication (Ngasala et al. 2015). Previous study by Swai and Schoonman (2011) in Tanga had a similar observation. Furthermore, other studies in Zimbabwe, Tanzania and Ghana reported that unhygienic practices along milk value chain predisposed milk to high bacterial load (Gran et al. 2002; Omore et al. 2009; Ngasala et al. 2015; Massawe et al. 2018; Lugamara et al. 2023). However, in this study, there was no correlation between the high bacterial load in milk and the unhygienic practices that were observed. Further, the poor hygienic practices were also realised during distribution of and handling of milk by vendors and restaurants/kiosks operators as previously reported by Kivaria et al. (2006) and Ngasala et al. (2015). This probably was the reason for majority of them had experienced losses due to spoiled milk.

The current study observed high bacteria load in milk, which authenticates the degree level of hygiene, practiced in the whole milk production process. The results of the present study showed a mean TBC of 5.3 log10 cfu/mL with more counts reported in milk from vendors ranging from 4.6 to 6.1 log10 cfu/mL. According to the East African community standards of raw cow milk (EAC 67:2007), the mean TBC of milk from farmers and vendors were above the required standard implying poor microbiological quality. The presence of such high bacterial load in milk may not be surprising since the raw milk harvested from dirty cows; dirty animal houses, the unhygienic environment and general milk handling may have contaminated the raw milk. The results of this study are inline to those done elsewhere in Tanzania in which most of the samples tested had higher bacterial count above standards. (Kweka 2002, Kivaria et al. 2006; Rwehumbiza et al. 2011; Ngasala et al. 2015; Massawe et al. 2018). Milk contaminated with bacteria has a short shelf life, aesthetically is not acceptable and can predispose the public to milk‐borne diseases.

The mean TCC recorded was 4.3 (log10 cfu/mL) with more counts recorded in vendors which ranged from 3.3 to 5.4 (log10 cfu/mL). Meanwhile according to East African community standards for CPC of raw milk (EAC 67:2007), good quality raw cow milk should not exceed CPC of 3 (log10 cfu/mL). In reference to this limit, it further indicates unhygienic handling of milk. Coliforms are used as indicator microorganisms and their presence implies a risk that other enteric pathogens like *Salmonella* may be present in the milk. The presence of coliforms therefore indicates a safety risk, and the numbers should therefore be of the minimum recommended levels in milk and milk products. Generally poor along the milk production and supply chain enhances contamination by coliforms. Cow dugs, personnel, water and dirty milking environment are the sources of coliform bacteria in milk (Oliver et al. 2005; Rwehumbiza et al. 2011; Ngasala et al. 2015; Massawe et al. 2018).

Surprisingly, the average levels of contamination in raw and pasteurised milk (5.3 log10 cfu/mL and 4.9 log10 cfu/mL respectively, for TBC, and 4.3 log10 cfu/mL and 3.6 log10 cfu/mL, respectively, for TCC) were not significantly different (*p* > 0.05). This finding concurs with other studies elsewhere (Hempen et al. 2004; Silva et al. 2009). The unsatisfactory quality of pasteurised milk observed in the current study is the consequence of the poor quality of raw milk used and/or a high level of recontamination after pasteurisation. Poor handling and storage of pasteurised milk in restaurants/kiosks observed during this study gave high possibilities for post boiling contamination. These findings highlight the fact that pasteurised milk of such poor microbiological quality poses a health threat to consumers.

The prevalence of *B. abortus* was 42.5% suggesting that there is high contamination rate as reported in other studies in Tanzania (Weinhäupl et al. 2000; Swai and Schoonman 2011; Karimuribo et al. 2007; Chitupila et al. 2015; Assenga et al. 2015; Mengele et al. 2023). More of the samples had come from farmers implying that the infection had originated from the cattle. However, it should be noted that the milk was pooled from a bulk collection hence the findings could not reflect the status of individual cows. With such a high prevalence of brucellosis in milk poses a threat to milk consumers. Indeed, majority of people in Tanzania consume raw milk (Kurwijila et al. 1995; Ngasala et al. 2015; Massawe et al. 2018). Pastoralists and agropastoralists have a preference of drinking raw fresh milk as they traditionally believe that it is healthier compared to pasteurised milk. On the other hand, pastoralists and agropastoralists ferment or clots raw milk for family use thinking that pasteurised milk does not give a good quality fermented or clotted milk. These practices increase chances of contracting *B. abortus* and other milk‐borne diseases. Such high infection rates of brucellosis may be linked with limited availability of livestock extension services and diseases control measures.

All the tested samples for *E. coli* O157:H7 showed negative results. This may show that the bacterium is not present in the cows in the study areas or the milk which was sampled was not contaminated with *E. coli* O157:H7. Previous study by Swai and Schoonman (2011) also did not isolate *E. coli* O157:H7 in milk. The study by Ndalama et al. (2013) reported negative results of *E. coli* O157:H7 in all tested meat in Dar es Salaam, Tanzania. This is in consistent with the fact that no outbreak related to food poisoning caused by *E. coli* 0157:H7 has been reported in Tanzania. Nonetheless, Lupindu et al. (2014) reported a prevalence of 1.6% of *E. coli* O157:H7 in cattle manure in Morogoro, Tanzania. Elsewhere in Ghana, Addo et al. (2011) reported negative results in all 250 milk samples tested. However, Omore et al. (2001) isolated *E. coli* O157:H7 in 1% of the samples in milk marketing survey in the Kenyan highlands. Similarly, Kang'ethe et al. (2007) isolated *E. coli* O157:H7 from cattle faeces in urban and peri‐urban settings of Nairobi, Kenya. Further work is recommended to elucidate the status of *E. coli* O157:H7 in milk and other food of animal origin in Tanzania.

### 4.1 Limitation of the Study

This study was a cross‐sectional design that milk samples from smallholder dairy farmers and milk retailers along with were administered structured questionnaires. This was done in two district councils out of 184 district councils of Tanzania. The results of this study represent the picture of what is happening in other places in Tanzania with similar livestock production system and milk handling practices, nonetheless, the sample size and differences in geographical locations and seasonality may differ from place to place. Further, the study analysed *B. abortus* in 87 out of 166 milk samples because of shortage of reagents, however results show the status of *Brucella* contaminations in milk from Lushoto and Handeni districts.

## 5 Conclusion

Generally, low poor cattle husbandry and unsanitary milking and post‐milking handling along the supply chain may have contributed to the high levels of microbial contamination in milk in the districts of Lushoto and Handeni. Since raw milk is an important vehicle for transmission of zoonoses and other pathogens, detection of *B. abortus* at higher prevalence poses health risks to the public. The Government, farmers and other players in the milk supply chain ought to have to take necessary measures to curb the problems.

## Author Contributions

Conceptualising of research idea, writing of proposal, data collection and drafting of manuscript—F.S. and H.E.N. Supervision of the whole research, data analysis and interpretation, review and perfection of manuscript—F.S. and H.E.N. Both the authors have read and agreed to the published version of the manuscript.

## Funding

This study is funded by Federal Ministry for Economic Cooperation and Development, Germany through the Safe Food, Fair Food project.

## Ethics Statement

Research permits were provided by the Vice Chancellor Sokoine University of Agriculture and permission letters were obtained from Executive Directors of Lushoto and Handeni districts. Verbal consent was obtained from each respondent after explaining the purpose and importance of the study prior to commencement of interviews and sampling. Participation in the study was on voluntary basis. All the information collected from the participants and the laboratory results obtained after milk samples analysis were kept under the custody of the researcher as confidential.

## Conflicts of Interest

The authors declare that they have no conflict of interest.

## Data Availability

The data generated through this study are contained in the manuscript. Additional data sets are available upon reasonable request.

## References

[vms371034-bib-0001] Abdel‐Rady, A. , and D. Sayed . 2009. “Epidemiological Studies on Subclinical Mastitis in Dairy Cows in Assiut Governorate.” Veterinary World 2, no. 10: 373–380.

[vms371034-bib-0002] Addo, K. K. , G. I. Mensah , K. G. Anning , et al. 2011. “Microbiological Quality and Antibiotic Residues in Informally Marketed Raw Cow Milk Within the Coastal Savannah Zone of Ghana.” Tropical Medicine and International Health 16, no. 2: 227–232. 21070512 10.1111/j.1365-3156.2010.02666.x

[vms371034-bib-0003] Aiello, S. E. , and M. A. Moses . 2006. The Merck Veterinary Manual, 11th Edition, Whitehouse Station, NJ: Merck & Co., Inc. 3325.

[vms371034-bib-0004] Anga, M. E. , F. Mgaya , B. Wadugu , E. M. Niccodem , and M. Matee . 2024. “Etiology of Febrile Illness Among Patients Seeking Care at a District Hospital in Manyara, Tanzania.” German Journal of Microbiology 4, no. 1: 6–14. 10.51585/gjm.2024.1.0029.

[vms371034-bib-0005] Ashraf, A. , and M. Imran . 2020. “Causes, Types, Etiological Agents, Prevalence, Diagnosis, Treatment, Prevention, Effects on Human Health and Future Aspects of Bovine Mastitis.” Animal Health Research Reviews 21, no. 1: 36–49. 10.1017/S1466252319000094. 32051050

[vms371034-bib-0006] Assenga, J. A. , L. E. Matemba , S. K. Muller , J. J. Malakalinga , and R. R. Kazwala . 2015. “Epidemiology of *Brucella* Infection in the Human, Livestock and Wildlife Interface in the Katavi‐Rukwa Ecosystem.” Tanzania. BMC Veterinary Research 11: 189. 26253151 10.1186/s12917-015-0504-8 PMC4529704

[vms371034-bib-0007] Cash‐Goldwasser, S. , M. J. Maze , M. P. Rubach , et al. 2018. “Risk Factors for Human Brucellosis in Northern Tanzania.” American Journal of Tropical Medicine and Hygiene 98: 598–606. 29231152 10.4269/ajtmh.17-0125 PMC5929176

[vms371034-bib-0008] Chitupila, G. Y. , E. V. G. Komba , and N. J. Mtui‐Malamsha . 2015. “Epidemiological Study of Bovine Brucellosis in Indigenous Cattle Population in Kibondo and Kakonko Districts, Western Tanzania.” Livestock Research for Rural Development 27, no. 6: 1–12.

[vms371034-bib-0009] Crump, J. A. , A. B. Morrissey , W. L. Nicholson , R. F. Massung , R. A. Stoddard et al. 2013. “Etiology of Severe Non‐Malaria Febrile Illness in Northern Tanzania: A Prospective Cohort Study.” PLOS Neglected Tropical Diseases 7, no. 7: e2324. 10.1371/journal.pntd.0002324. 23875053 PMC3715424

[vms371034-bib-0010] De Buyser, M. L. , B. Dufour , M. Maire , and V. Lafarge . 2001. “Implication of Milk and Milk Products in Food‐Borne Diseases in France and in Different Industrialized Countries.” International Journal of Food Microbiology 67: 1–17. 11482557 10.1016/s0168-1605(01)00443-3

[vms371034-bib-0011] East African Community . 2007. East African Standard, Raw Cow Milk Specification, 2. East African Community.

[vms371034-bib-0012] East African Community (EAC) . 2006. East African Standard, Raw Cow Milk Specification, 1–2. East African Community.

[vms371034-bib-0013] Eshitera, E. E. , J. O. Onono , G. Oluga , P. Gathura , and W. E. Mwihia . 2023. “Milk Value Chains Maps Identifying Challenges and Vulnerabilities in the Pastoral and Agro‐Pastoral Areas of Narok, Kenya.” East African Journal of Science, Technology and Innovation 4, no. 2: 1–15. 10.37425/eajsti.v4i2.610.

[vms371034-bib-0014] Flint, J. , Y. Duynhoven , F. Angulo , et al. 2005. “Estimating the Burden of Acute Gastroenteritis, Food‐Borne Diseases and Pathogens Commonly Transmitted by Food.” Journal of Clinical Infectious Diseases 41: 698–704. 16080093 10.1086/432064

[vms371034-bib-0015] Gran, H. M. , A. N. Mutukumira , A. Wetlesen , and J. A. Narvhus . 2002. “Smallholder Dairy Processing in Zimbabwe: Hygiene Practices During Milking and the Microbiological Quality of the Milk at the Farm and on Delivery.” Food Control 13: 41–47.

[vms371034-bib-0016] Gwandu, S. H. H. E. Nonga R. H. Mdegela A. S. Katakweba T. S. Suleiman , and R. Ryoba . 2018. “Assessment of Raw Cow Milk Quality in Smallholder Dairy Farms in Pemba Island Zanzibar, Tanzania.” Veterinary Medicine International 12, no. 2018: 1–9. 10.1155/2018/1031726. PMC5867610 29721257

[vms371034-bib-0017] Harley, J. P. 2013. Laboratory Exercises in Microbiology. McGraw‐Hill.

[vms371034-bib-0018] Hempen, M. , F. Unger , S. Münstermann , M. Seck , and V. B. Niamy . 2004. “The Hygienic Status of Raw and Sour Milk From Smallholder Dairy Farms and Local Markets and Potential Risk for Public Health in The Gambia, Senegal and Guinea.” Animal Health Research Working Paper International Trypanotolerance Centre. pp 1–54.

[vms371034-bib-0019] Hosseinzadeh, S. , and H. D. Saei . 2014. “ *Staphylococcal* Species Associated With Bovine Mastitis in the Northwest of Iran: Emerging of Coagulase‐Negative Staphylococci.” International Journal of Veterinary Science and Medicine 2, no. 1: 27–34. 10.1016/j.ijvsm.2014.02.001.

[vms371034-bib-0020] ISO/FDIS . 2001. Milk and Milk Products—General Guidance for the Preparation of Samples, Initial Suspensions and Decimal Dilutions for Microbiological Examination. International Organization for Standardization.

[vms371034-bib-0021] ISO 7218 . 2007. Microbiology of Food and Animal Feeding Stuffs—General Requirements and Guidance for Microbiological Examination . International Organization for Standardization.

[vms371034-bib-0022] Kang'ethe, E. K. , J. O. Onono , B. MacDermott , and S. M. Arimi . 2007. “Isolation of *E. coli* O157:H7 From Milk and Cattle Faeces From Urban Dairy Farming and Nondairy Farming Neighbour Households in Dagoretti Division, Nairobi, Kenya: Prevalence and Risk Factors.” East African Medical Journal 84: 65–75. 10.4314/eamj.v84i11.9578 18338724

[vms371034-bib-0023] Karimuribo, E. D. , H. A. Ngowi , E. S. Swai , and D. M. Kambarage . 2007. “Prevalence of Brucellosis in Crossbred and Indigenous Cattle in Tanzania.” Livestock Research for Rural Development 19: Article #148. http://www.lrrd.org/lrrd19/10/kari19148.htm.

[vms371034-bib-0024] Katakweba, A. A. S. , M. M. A. Mtambo , J. E. Olsen , and A. P. Muhairwa . 2012. “Awareness of human Health Risks Associated With the Use of Antibiotics Among Livestock Keepers and Factors That Contribute to Selection of Antibiotic Resistance Bacteria Within Livestock in Tanzania.” Livestock Research for Rural Development 24: Article #170. http://www.lrrd.org/lrrd24/10/kata24170.htm.

[vms371034-bib-0025] Katale, B. Z. , E. V. Mbugi , E. D. Karimuribo et al. 2013. “Prevalence and Risk Factors for Infection of Bovine Tuberculosis in Indigenous Cattle in the Serengeti Ecosystem.” Tanzania. BMC Veterinary Research 9, no. 1: 267. 24377705 10.1186/1746-6148-9-267 PMC3881215

[vms371034-bib-0026] Katale, B. Z. , E. V. Mbugi , S. Kendal , et al. 2012. “Bovine Tuberculosis at the Human‐Livestock‐Wildlife Interface: Is It a Public Health Problem in Tanzania? A Review.” Onderstepoort Journal of Veterinary Research 79, no. 2: 1–8. Art. #, 8 pages 463. 10.4102/ojvr.v79i2.463. 23327384

[vms371034-bib-0027] Katjiuongua, H. , and S. Nelgen . 2014. Tanzania Smallholder Dairy Value Chain Development: Situation Analysis and Trends ILRI Project Report. ILRI.

[vms371034-bib-0028] Kivaria, F. M. , J. P. T. M. Noordhuizen , and A. M. Kapanga . 2006. “Evaluation of the Hygienic Quality and Associated Public Health Hazards of Raw Milk Marketed by Smallholder Dairy Producers in the Dar es Salaam Region, Tanzania.” Tropical Animal Health Production 38: 185–194. 16986766 10.1007/s11250-006-4339-y

[vms371034-bib-0029] Kothari, C.R. (Eds.). 2004. Research Methodology, Method and Techniques, 175–180. New Age International Publishers.

[vms371034-bib-0030] Kurwijila, R. L. , N. Mdoe , D. N. Nyange , R. M. Auerbock , and H. N. Malya . 1995. Assessment of Fresh Milk and Milk Products and Consumption in Dar es Salaam, Report to the Austro Project Association, 54. Austro Project Association, Dar es Salaam.

[vms371034-bib-0031] Kweka, L. A. 2002. “Quality and Antibiotic Residues in Milk Obtained in Tanga, Tanzania.” MSc Diss. (Unpublished), Sokoine University of Agriculture, 1–96.

[vms371034-bib-0032] Lugamara, C. B. 2024. “Post‐Harvest Milk Losses Among Tanzanian Milk Supply Chain Actors and Its Implications on Milk Producing Households′ Food Security.” PhD Thesis, Sokoine University of Agriculture, 234.

[vms371034-bib-0033] Lugamara, C. B. , J. K. Urassa , and G. D. Massawe . 2023. “A Review of Post‐Harvest Milk Losses in Tanzania's Milk Sector: Lessons From Production to Consumption.” Tanzania Journal of Agricultural Sciences 22, no. 02: 1–8.

[vms371034-bib-0034] Lunogelo, H. B. , M. F. Songora , and J. Lasway . 2021. “Innovation and Inclusive Industrialisation in Agro Processing: The Tanzanian Dairy Value Chain.” Working Paper v1 for the Innovation and Inclusive Industrialization in Agro‐Processing Project (IIAP). Economic and Social Research Foundation.

[vms371034-bib-0035] Lupindu, A. M. , J. E. Olsen , H. A. Ngowi , et al. 2014. “Occurrence and Characterization of Shiga Toxin‐Producing *Escherichia coli* O157:H7 and Other Non‐Sorbitol‐Fermenting *E. coli* in Cattle and Humans in Urban Areas of Morogoro, Tanzania.” Vector Borne Zoonotic Diseases 14, no. 7: 503–510. 10.1089/vbz.2013.1502. 24901881

[vms371034-bib-0036] Makoye, M. , and H. E. Nonga . 2026. “Occurrence and Public Health Implications of *Brucella Abortus* and Antimicrobial Residues in Raw Cow Milk in Bukombe District, Tanzania.” Veterinary Medicine and Science 12: e70944. 10.1002/vms3.70944. 41954297 PMC13063390

[vms371034-bib-0037] Mashauri, H. L. B. L. Max S. M. Makweba , and H. E. Nonga . 2025. “Antimicrobial Residues and Food Safety: A Public Health Crisis of Concern in Tanzania.” Health Science Reports 8, no. 8: e71155. 40799980 10.1002/hsr2.71155 PMC12339913

[vms371034-bib-0038] Massawe, H. F. , R. H. Mdegela , and L. R. Kuwrijila . 2018. “Association of Smallholder Dairy Farmers Management and Milking Practices With Bacterial Quality of Milk in Mbeya, Tanzania.” Bulletin of Animal Health and Production in Africa 66: 51–65.

[vms371034-bib-0039] Mbiha . 2008. “Analysis of the Dairy Value Chain in the Dar Es Salaam Milk Shed, Tanzania.” MSc Diss., Sokoine University of Agriculture, 151.

[vms371034-bib-0040] Mdegela, R. H. , E. R. Mwakapeje , B. Rubegwa , et al. 2021. “Antimicrobial Use, Residues, Resistance and Governance in the Food and Agriculture Sectors, Tanzania.” Antibiotics 10: 454. 10.3390/antibiotics10040454. 33923689 PMC8073917

[vms371034-bib-0041] Mengele, I. J. , G. M. Shirima , B. M. Bronsvoort , L. E. Hernandez‐Castro , and E. A. J. Cook . 2023. “Diagnostic Challenges of Brucellosis in Humans and Livestock in Tanzania: A Thematic Review.” CABI One Health. 1–16. 10.1079/cabionehealth.2023.0001.

[vms371034-bib-0042] MLF . 2023. “Ministry of Livestock and Fisheries Budget Speech.” https://www.mifugouvuvi.go.tz/.

[vms371034-bib-0043] Mpatswenumugabo, J. P. , M. A. Mukasafari , J. B. Ndahetuye , E. Wredle , and R. Båge . 2023. “A Systematic Literature Review of Milk Consumption and Associated Bacterial Zoonoses in East Africa.” Journal of Applied Microbiology 134, no. 4: lxad080. 10.1093/jambio/lxad080. 37081784

[vms371034-bib-0044] Msalya, G. 2017. “Contamination Levels and Identification of Bacteria in Milk Sampled From Three Regions of Tanzania: Evidence From Literature and Laboratory Analyses.” Veterinary Medicine International 2017: 1–10. 10.1155/2017/9096149. PMC5602642 28948059

[vms371034-bib-0045] Nancy, P. R. , J. Geert , A. Marina , R. Rudi , and M. F. H. Lieve . 1996. “Direct Detection of *Brucella* Spp. in Raw Milk by PCR and Reverse Hybridization With 16S‐23S rRNA Spacer Probes.” Journal of Applied Environmental Microbiology 62, no. 5: 1683–1688. 10.1128/aem.62.5.1683-1688.1996 PMC167942 8633866

[vms371034-bib-0046] Ndalama, E. 2013. “Assessment of Hygienic Practices and Faecal Contamination of Beef at Vingunguti Slaughterhouse in Dar es salaam, Tanzania.” Unpublished Research Paper for Award of MPVM Degree, Sokoine University of Agriculture, 1–29.

[vms371034-bib-0047] Ngasala, J. B. , H. E. Nonga , and M. M. A. Mtambo . 2015. “Assessment of Raw Milk Quality and Stakeholders′ awareness on Milk‐Borne Health Risks in Arusha City and Meru District, Tanzania.” Tropical Animal Health and Production 47, no. 5: 927–932. 25863955 10.1007/s11250-015-0810-y

[vms371034-bib-0048] Ogutu, C. , L. Kurwijila , and A. Omore . 2014. “Review of Successes and Failures of Dairy Value Chain Development Interventions in Tanzania.” More Meat, Milk and Fish by and for Poor Programme, CGIAR Centres. http://livestockfish.cgiar.org.

[vms371034-bib-0049] Oliver, S. P. , B. M. Jayarao , and R. A. Almeida . 2005. “Foodborne Pathogens in Milk and the Dairy Farm Environment: Food Safety and Public Health Implications.” Foodborne Pathogens and Disease 2, no. 2: 115–129. 15992306 10.1089/fpd.2005.2.115

[vms371034-bib-0050] Omore, A. , S. Staal , L. Kurwijila , G. Aning , N. Mdoe , and G. Nurah . 2001. Indigenous Market for Dairy Products in Africa: Trade‐Offs Between Food Safety and Economics. Proceedings of Symposiums on Dairy Development in the Tropics, 19–24. Utrecht University.

[vms371034-bib-0051] Omore, A. O. , S. J. Staal , F. Wanyoike , et al. 2009. “Market Mechanisms and Efficiency in Urban Dairy Products Markets in Ghana and Tanzania.” ILRI Research Report 19, International Livestock Research Institute, 51.

[vms371034-bib-0052] Rwehumbiza, J. M. , R. Ryoba , and E. D. Karimuribo . 2011. “Assessment of Microbiological Status and Presence of Antibiotic Residues in Cow Milk Produced in Bagamoyo and Kisarawe Districts, Tanzania.” Tanzania Veterinary Journal 28: 60–69.

[vms371034-bib-0053] Sangeda, R. Z. , A. Baha , A. Erick , et al. 2021. “Consumption Trends of Antibiotics for Veterinary Use in Tanzania: A Longitudinal Retrospective Survey from 2010–2017.” Frontiers Tropical Diseases 2: 1–11.

[vms371034-bib-0054] Schoder, D. , A. Maichin , B. Lema , and J. Laffa . 2013. “Microbiological Quality of Milk in Tanzania: From Maasai Stable to African Consumer Table.” Journal of Food Protection 76, no. 11: 1908–1915. 10.4315/0362-028X.JFP-13-101. 24215695

[vms371034-bib-0055] Silva, R. , A. G. Cruz , A. F. J. Faria , et al. 2009. “Pasteurized Milk: Efficiency of Pasteurization and Its Microbiological Conditions in Brazil.” Foodborne Pathogens and Disease 7, no. 2: 217–219. 10.1089/fpd.2009.0332 19785537

[vms371034-bib-0056] Sivapalasingams, S. , C. R. Friedman , L. Cohen , and R. V. Tauxe . 2004. “Fresh Produce: A Growing Cause of Outbreaks of Foodborne Illness in the United States.” Journal of Food Protect 67, no. 10: 2342–2353. 10.4315/0362-028x-67.10.2342 15508656

[vms371034-bib-0057] Swai, E. S. , and L. Schoonman . 2011. “Microbial Quality and Associated Health Risks of Raw Milk Marketed in the Tanga Region of Tanzania.” Asian Pacific Journal of Tropical Biomedicine 1, no. 3: 217–222. 10.1016/S2221-1691(11)60030-0. 23569762 PMC3609189

[vms371034-bib-0058] The Animal Diseases Act Cap 156 . 2003. https://www.mifugouvuvi.go.tz/.

[vms371034-bib-0059] The Dairy Industry Act Cap 262 . 2006. https://www.mifugouvuvi.go.tz/.

[vms371034-bib-0060] The Public Health Act, Cap 263 . 2009. https://www.parliament.go.tz/polis/uploads/bills/acts/1452146412‐ActNo‐1‐2009.pdf.

[vms371034-bib-0061] The Standards Act Cap 130 . 2009. https://www.tbs.go.tz/publications/23.

[vms371034-bib-0062] The Tanzania Food and Nutrition Act Cap 109 . 2009. https://tanzanialaws.com/t/364tanzania‐food‐and‐nutrition‐act.

[vms371034-bib-0063] The Tanzania Food Drugs and Cosmetics Act . 2003. https://www.tmda.go.tz/.

[vms371034-bib-0064] The Veterinary Act Cap 319 . 2003. https://www.mifugouvuvi.go.tz/.

[vms371034-bib-0065] TLMP . 2017. “Tanzania Livestock Master Plan of 2017/2018–2021/2022.” https://www.mifugouvuvi.go.tz/.

[vms371034-bib-0066] TZS . 2007. Microbiology of food and Animal Feeding stuffs—Method for Enumeration of Microorganisms—Colony Count Technique at 30°C. Tanzania Bureau of Standards.

[vms371034-bib-0067] Ukita, M. , N. Hozé , T. Nemoto , et al. 2021. “Quantitative Evaluation of the Infection Dynamics of Bovine Brucellosis in Tanzania.” Preventive Veterinary Medicine 194: 105425. 10.1016/j.prevetmed.2021.105425. 34256237

[vms371034-bib-0068] UNDP/BCS/TetraPak . 2006. “A Value Chain Analysis and Socio‐Economic Assessment of the Dairy Industry in Tanzania.” In *Proceedings of the 6th National Dairy Development Conference*, 19–32. Tanzania.

[vms371034-bib-0069] Wang, R. F. , W. W. Cao , and C. E. Cerniglia . 1997. “A Universal Protocol for PCR Detection of 13 Species of Foodborne Pathogens in Foods.” Journal of Applied Microbiology 83: 727–736. 9449811 10.1046/j.1365-2672.1997.00300.x

[vms371034-bib-0070] Weinhäupl, I. , K. C. Schöpf , D. Khaschab , A. M. Kapaga , and H. M. Msami . 2000. “Investigations on the Prevalence of Bovine Tuberculosis and Brucellosis in Dairy Cattle in Dar es Salaam Region and in Zebu Cattle in Lugoba Area, Tanzania.” Tropical Animal Health and Production 32, no. 3: 147–154. 10907285 10.1023/a:1005231514467

